# Perplexing Role of P-Glycoprotein in Tumor Microenvironment

**DOI:** 10.3389/fonc.2020.00265

**Published:** 2020-03-05

**Authors:** Kianna Robinson, Venkataswarup Tiriveedhi

**Affiliations:** ^1^Department of Biological Sciences, Tennessee State University, Nashville, TN, United States; ^2^Department of Pharmacology, Vanderbilt University, Nashville, TN, United States

**Keywords:** P-glycoprotein, cancer, tumor immunology, chemotherapy, metastasis

## Abstract

Development of multidrug resistance (MDR) still remains a major obstacle to the long-term success of cancer therapy. P-glycoprotein (P-gp) is a well-identified membrane transporter with capability to efflux drug molecules out of the cancer cell leading to reduced efficiency of chemotherapy. Cancer cells upregulate P-gp expression as an adaptive response to evade chemotherapy mediated cell death. While several P-gp inhibitors have been discovered by *in silico* and pre-clinical studies, very few have successfully passed all phases of the clinical trials. Studies show that application of P-gp inhibitors in cancer therapy regimen following development of MDR achieved limited beneficial outcomes. While, the non-specific substrate binding to P-gp has made the drug-design a challenge, a bigger perplexing challenge comes from its role in tumor immunology. Expression of P-gp was noted immune cell phenotypes with apparently antagonistic functionality. Both pro-tumor MΦ2-macrophages and, anti-tumor NK-cell and Th17/CD4^+^T cell subsets have shown enhanced expression of P-gp. While drug based inhibition of P-gp in pro-tumor immune cell phenotypes could promote tumor elimination, however, it would not be a rational choice to exert inhibition of P-gp on anti-tumor immune cell phenotypes. This mutually exclusive paradigm of P-gp functionality requires a more comprehensive and detailed understanding of its role in tumor microenvironment with active interplay of cancer and immune cells in the tumor *mileu*. In this review, we focus on the current understanding of the role of P-gp in cancer cells and immune cells and finally attempt to highlight some caveats in the current understanding of its role in comprehensive tumor microenvironment along with challenges in the development of P-gp inhibitors toward anti-cancer therapy.

## Introduction

Multidrug resistance (MDR) accounts for chemotherapeutic resistance in cancer cells (1). Three major proteins namely P-glycoprotein (P-gp, also referred to as MDR1), MDR-associated protein 1 (MRP1) and breast cancer resistance protein (BCRP), were shown to play a critical in MDR (2). These three proteins belong to a family of 48 energy-dependent membrane transporter proteins called adenosine triphosphate (ATP)-binding cassette (ABC) efflux pumps (3, 4). This group of ABC transporters have a diverse epithelial cell surface expression including on gastrointestinal tract, hepatobiliary tract, renal tubules, adrenal cortex, placenta, and blood-brain barrier membranes (5). Under physiological conditions, ABC transporters are involved in efflux of lipids, sterols, small microbial peptides and toxins out of the cytoplasm (6). P-gp is most studied and well-characterized MDR transporter associated with resistance to cancer chemotherapy (7). Szackas et al. have previously tested 118 compounds with known putative mechanism of action on NCI-60 cancer cell lines (8). Their results have demonstrated that more than 95% of the compounds exerted a negative correlation between drug sensitivity and P-gp expression. Compounds such as geldanamycin, paclitaxel and its taxane analogs, doxorubicin, vinblastine, and bisantrene demonstrated a striking negative correlation, while compounds such as hydroxyurea, methotrexate, and 5-fluorouracil were found to have been invariably non-correlated or slightly positively correlated with drug sensitivity index. While some cancer cells (such as melonama and renal cancers) have an enhanced genetic and epigenetic modulators causing higher constitutive expression of P-gp, majority of other solid tumors induce expression of P-gp as a tumor resistance response following initiation of chemotherapy (9). Several P-gp inhibitors have been studied to improve the chemotherapeutic susceptibility of solid tumors (10). However, majority of these clinical trials have failed due to several reasons, an important reason being the high drug doses need to exert P-gp transporter inhibition. Currently, several new drug discovery projects have an array of novel pro-drug compounds in pipeline to bypass or exert a more sustained P-gp inhibition (11). Interestingly, over the past two and half decade accumulating evidence suggested that the expression of P-gp in inflammatory immune cell subset (12–15). This could exert a potential anti-cancer cytotoxic functionality. However, a detailed understanding of this apparently contrasting role of P-gp in cancer and immune cells in the context of tumor microenvironment is yet to evolve. In this review, we will briefly describe the molecular details of P-gp and prevailing understanding on its inhibitors. We will than focus on the current immunological evidence of P-gp in various immune cell phenotypes with potential future insights on tumor immunotherapy.

## P-gp Genetics

The *p-gp/abcb1* gene is located on chromosome 7q21.12 and contains 29 exons in a genomic region spanning 209.6 kb. The messenger RNA (mRNA) is 4872 bp in length, including the 5′ untranslated region (RefSeq accession NM_000927.3), which is expressed into a 141 KDa protein with 1,280 amino acids (16). To date (as of Nov 2019, NCBI-SNP view database), in the coding region alone, upto 1,200 single nucleotide polymorphisms (SNPs) have been reported with varied impact on protein expression and functionality. Of these the three most studied SNPs in the protein coding region of P-gp are rs1045642 (3435T>C, Ile1145Ile), rs2032582 (2677T>G/A, Ser893Ala/Thr), and rs1128503 (1236T>C, Gly412Gly) (17). Further, while 28% of the SNPs were reported in the transmembrane domain 72% of the SNPs were reported in intra- and extracellular regions of P-gp.

The synonymous mutation, C to T transition at position 3435 (rs1045642, 3435T>C) results in an unaltered amino acid sequence (Ile1145Ile) and could be expected not to change the protein functionality (18). In general, the 3435C allele occurs at 34–90% frequency across all populations with high expression 3435CC genotype in Africans compared to Caucasians or Japanese (19–21). Although this is a synonymous mutation, interestingly, it is not generally considered a silent mutation. Hoffmeyer et al. have demonstrated that 3435 TT genotype population demonstrated lower expression of P-gp in the epithelial cells of digestive tract (22). The *3435C* allele showed higher mRNA transcript levels compared to the *3435T* allele (23). This differential gene expression level is considered to be due to instability in the mRNA secondary structure which requires more time for mRNA folding/unfolding during translation process resulting in altered membrane insertion and tertiary structural orientation and thus leading to variations in the substrate affinity. For these reasons, the *3435CC* genotype is correlated with a higher P-gp expression and function compared to either *3435CT* or *3435TT* genotypes (24, 25). In the context of tumor resistance, patients with *3435TT* genotype might be expect to develop minimal resistance to chemotherapy compared to *3435CC* genotype, and therefore requiring lower amount of drug for cancer cell elimination (25). Pharmacokinetic studies with cyclosporine have demonstrated that patients with 3435*TT* genotype had enhanced intracellular drug concentration compared to 3435*CC* genotype. Similarly, pharmacodynamic studies with tacrolimus and sirolimus have demonstrated that compared to 3435*CC* genotype, patients with 3435*TT* genotype had higher immunosuppression as evidenced by decreased circulating levels of inflammatory cytokine, interleukin-2 (IL-2) (26).

The rs2032582 SNP (2677T > G/A, Ser893Ala/ Thr), with three allelic variants, although well-studied have some discordant outcomes on the actual protein functionality. The frequency of 2677T allele coding for serine-893 varies as widely as 2–65% among various ethnicities (23). Interestingly, the frequency of homozygous 2677 GG genotype leading to 893-Ala/Ala P-gp is found to be as high as 81% in African populations, as compared to the frequency of only 10–32% in other demographics such as European, Mexican, Native America, Asian, and Indian populations. Along with these SNPs, another allele, 2677A bearing Thr-893 P-gp has been reported to be at lower frequency of only 0–17% across various populations. In spite of extensive studies on this non-synonymous mutation inducing SNP, the potential impact on the P-gp expression and functionality is unclear (27). The Ser-893 P-gp has shown to have apparently conflicting functional outcomes with all three (increase, decrease and no change) outcomes on the pharmacodynamics properties. Similarly, studies with Ala-893 vs. Ser-893 mutation have shown no difference in the treatment outcomes in inflammatory bowel diseases (Crohn's and ulcerative colitis) (28). Similarly, a third SNP, rs1128503 (1236T>C) bearing synonymous mutation leading to Gly412Gly P-gp is reported to have a wide frequency of 30 to 93% among various populations (29). However, the pharmacokinetic and pharmacodynamics differences between the genotypes, 1236CC/CT/TT, have not been confirmed (30).

## P-gp: Protein Expression, Structure, and Function

**Figure 1:**
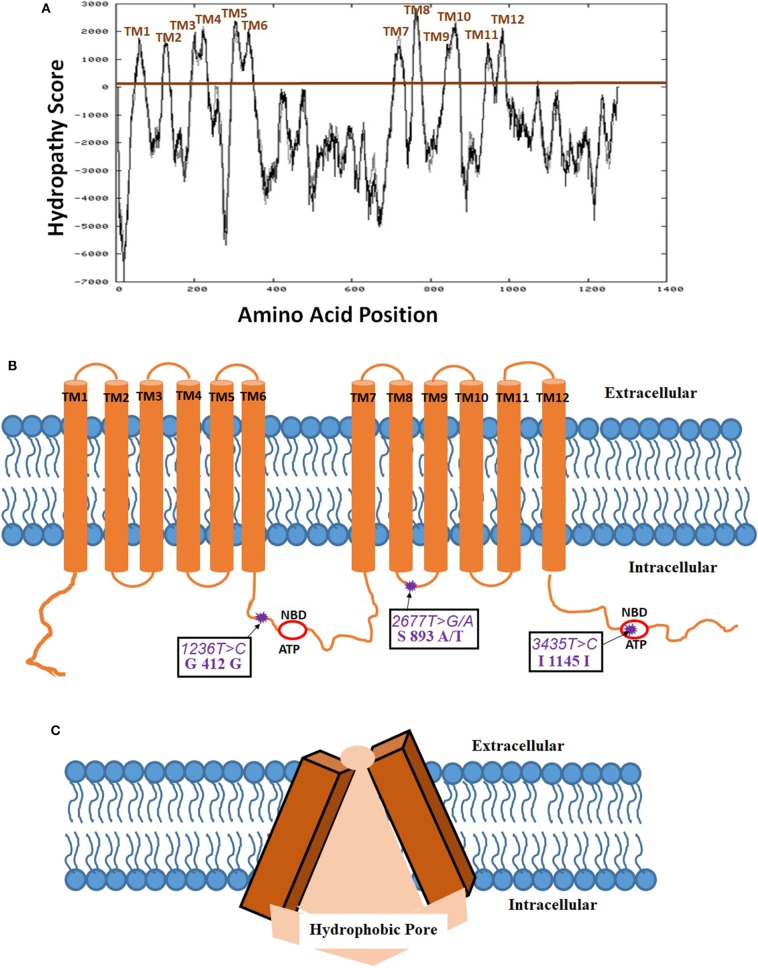
Membrane localization of P-glycoprotein (P-gp, PDB ID: 6QEX). **(A)** Kyte-Doolittle hydropathy plot determining the amino acid positions in the twelve transmembrane domains (TMD) of P-gp (https://embnet.vital-it.ch/cgi-bin/TMPRED_form_parser; EXPASy Bioinformatics resource portal); **(B)** Schematic of the membrane localization of 12 TMDs, 2 ATP- nucleotide binding domains (NBD), and 3 most common single nucleotide polymorphisms (SNP) on P-gp; **(C)** Tertiary three dimensional inverted V-shaped structure of P-gp.

Biedler et al. (31) for the first time suggested the potential existence of multidrug resistance (MDR) phenomena mediated by a cell surface protein (31). Riordan et al. (32) have first cloned the P-gp cDNA and expressed in mammalian cell lines to provide proof that MDR is indeed mediated by a membrane protein (32). Later, Schinkel et al. (33) using a murine *abcb1* (P-gp) knock-out model demonstrated a 100-fold increased brain tissue concentration of antiparasitic medication, ivermectin, in these genetically engineered mice (33). For constitutive expression of this gene, there seems to be two transcriptional start regions in the proximal promoter region of exon 1 and intron 1. The mRNA transcript for this gene compromising of 5' untranslated region is 4,872 bp long and is translated into 1,280 amino acid P-gp protein. The secondary structure has twelve transmembrane domains (TMD) as also evidence by Kyte-Doolittle hydropathy plot (Figure 1). Several alternative transcripts and splice variants with undetermined significance were reported in the literature, however, not discussed in the current review. P-gp is post-translationally modified by differential phosphorylation and *N*-glycosylation which is thought to impact its final functionality (34). The serine residues of P-gp, S661 and S683 are phosphorylated by PKC and PKA, respectively (35). Additionally, phosphorylation of S683 by Pim-1 selectively on glycosylated P-gp is considered to induce multimerization and surface membrane stabilization (36). While two phosphorylation residues were shown to bind with tubulin, it has not been shown to be important in inducing downstream signaling and protein functionality (37, 38). The 12 TMDs form a hydrophobic pore-like-channel in the cell membrane to promote drug efflux of hydrophobic and amphipathic compounds (Figure 1). The two ATP-binding domains are located in the cytoplasmic intracellular side of the protein. The first high-resolution X-ray crystallography structure at a resolution of 3.8 Å of mouse P-gp, which has 87% homology with human P-gp, was reported in 2009 (39). Further studies with slightly improved (up to 3.3 Å) resolution also showed predominantly similar tertiary structural features (40, 41). The tertiary structure of P-gp protein exhibits high membrane flexibility to allow for multiple three-dimensional (3D) reorientations, possibly playing an important functional role in binding and efflux of a wide array of drug substrates (42). Interestingly, *in silico* structure activity relationship (SAR) studies demonstrated that P-gp had the capacity to differentially bind with stereoisomers of the same compound and also has multiple binding sites to allow binding and efflux of multiple drug substrates (43). While the initial SAR studies with P-gp have been challenging mainly due to its high hydrophobicity index making it insoluble in water and high tertiary structural flexibility, more recent studies by Alam et al. revealed a 3.5- Å resolution structure using reconstituted in lipidic nanodiscs allowing for much better SAR biochemical understanding (44).

The structure of P-gp displays the canonical ABC transporter fold consisting of two pseudo-symmetric transmembrane domains, with each half containing six transmembrane helices (TM) and one cytosolic domain has ATP-nucleotide binding functionality (NBD). The two NBD domains in P-gp, which are largely conserved in many ABC proteins, dimerize to bind and hydrolyze ATP at the interface. A 60–70 amino acid flexible linker with several phosphorylation sites connects the two pseudo-halves of P-gp (45). The cytoplasmic side of the protein encloses a 6000 Å3 large cavity (39). Drugs are thought to enter this cavity for binding through portals open on the cytoplasm and the inner leaflet of the membrane and exit out through the extracellular side which generally has a 70–200 Å3 pore size depending on the protein orientation (Figure 1). P-gp undergoes dynamic conformational changes to allow for an array of substrate binding and efflux which is associated with ATP binding and hydrolysis on the cytoplasmic side allowing unidirectional outward flow of the substrates. Thermodynamic studies have demonstrated that while inward V-conformation is energetically-feasible conformation and transient outward facing conformation is adopted at high-energy state with the consumption of ATP-derived energy (46). In spite of the controversies in the reported crystal structure regarding the location of ATP-binding domain due to the use of detergents, and the absence of nucleotides to obtain the crystal structure, however, it is well-documented that the two ATP-binding domains should be on the intracellular side as the cellular concentration of ATP (1–10 mM) far exceeds the domain binding constant (~0.01 mM) (47, 48). Regardless of the controversies on the P-gp tertiary membrane bound structure, the resolved crystal structure enables the *in silico* identification of the drug substrates and inhibitors for P-gp.

**Figure 2:**
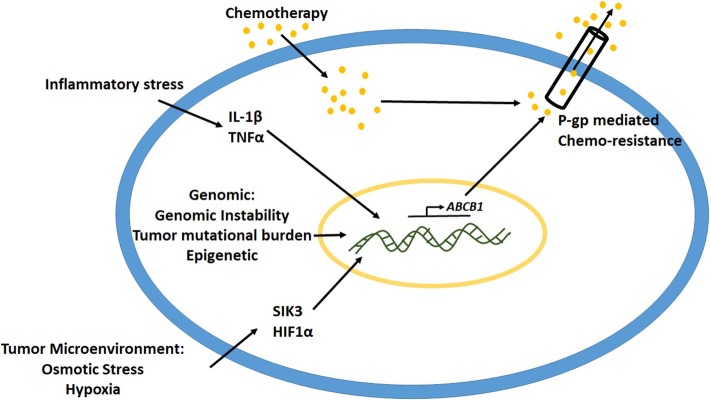
Mechanisms leading to upregulation of P-gp expression.

Several causes such as intrinsic cancer genomic instability, epigenetic mechanisms and inflammatory stressors in the tumor microenvironment have been implicated to play a critical role in the upregulation of P-gp expression (Figure 2) (49). Studies have demonstrated that gene rearrangements and tumor mutational burden are important mechanisms to control and modulate promoter region of *abcb1* gene leading to its expression (50, 51). Oncogenes such as Ras, p53, c-Raf, etc. have been associated with the regulation of P-gp expression (52, 53). In various kinds of leukemias with enhanced P-gp expression, the promoter region of the gene was shown to be demethylated, suggesting the role of epigenetic modification toward activation of P-gp mediated drug resistance (9, 54). Studies have shown that, following cancer chemotherapy, there is an upregulation of acetyl-H3 and histone deacetylase activity (55, 56). The acetyl-H3 was shown to act at 968 bp upstream P-gp gene in the promoter region. Transcription factors such as CEBPβ have been shown to induce P-gp expression in MCF-7 breast cancer cell lines (57). Previous studies from our laboratory demonstrated that high salt-mediated osmotic stress (Δ0.05 mM NaCl) on MCF-7 and MDA-MB-231 breast cancer cells enhanced intracytoplasmic calcium concentration through activation of store operated calcium entry (SOCE) from endoplasmic reticulum (58). This hypertonic stress induced P-gp expression leading to paclitaxel drug resistance in these breast cancer cells. Further, our murine tumor studies demonstrated that orthotopic breast tumors with MCF-7 cells pre-cultured in hypertonic stress conditions, exerted higher tumor progression kinetics compared to the basal media cultured counterpart. These data suggest that osmotic stress in most solid organ tumors in itself induces P-gp mediated drug resistance.

Studies from some laboratories have demonstrated that hypoxic stress enhances P-gp expression through interaction of HIF1α to P-gp promoter region (59). Sakata et al. have previous demonstrated that cells which developed hypoxia mediated drug resistance did not demonstrate significant increase in the mRNA of P-gp, thus suggesting that the mechanism of hypoxia mediated chemoresistance is different from P-gp pathway (60). Similarly, P-gp-independent MDR was also reported in osteosarcoma cells. Avnet et al. have demonstrated that short term changes in extracellular acidosis induced a reversal plasma membrane pH gradient along with decreased intracellular concentration of drugs such as doxorubicin and cisplatin, with no change P-gp functionality, thus suggesting a non-P-gp mediated MDR (61). Therefore, along with P-gp, other factors in the tumor microenvironment could also play a significant role in MDR.

### P-gp Substrate and Drug Interactions

A large variety of molecules with divergent chemical structures (cyclic, linear, polar, non-polar, linear-hydrophobic, aromatic) and molecular weights (from 250 to 4,000 Da) are known to efflux through P-gp transporter (Table 1) (62). cyclosporine-A and verapamil were some of the first identified competitive inhibitors to P-gp. Crystallograhy studies (based on mouse P-gp) have shown that drug-binding motif in the inward V-orientation of P-gp is made up of both hydrophobic (and aromatic) residues to facilitate hydrophobic and van der Waals interactions, and also has few polar side chains (e.g., Q343, Q721, Q942, Q986, and S975) to facilitate the formation of hydrogen-bonds with ligands, thus explaining for the diverse substrate and inhibitor binding to P-gp (63, 64). A recent high-throughput screen of 10,804 compounds by Lee et al. has identified a total of 90 substrates of which 55 were novel. Among these, substrates for P-gp included anti-cancer small molecules such gedatolisib (PKI-587, phosphoinositide 3-kinase/mammalian target of rampamycin inhibitor), AT7159 (cyclin-dependent kinase inhibitor), AT9283 (Janus kinase 2/3 inhibitor), and ispinesib (kinesin spindle protein inhibitor) (65). Currently, although there is a lot of interest in the development of small molecule inhibitors of P-gp, to overcome multi-drug resistance (MDR) in cancer chemotherapy, this enthusiasm is curtailed by the fact that majority of previously discovered inhibitors could not succeed in passing FDA approved clinical phase trials. There are several reasons for this failure to pass clinical trials, but a major reason is that the tissue toxicity of the drugs at the high dose needed for P-gp inhibition (66). For example, verapamil and cyclosporine-A, some of the first discovered P-gp inhibitors tested in clinical trials, demonstrated low-affinity to P-gp requiring several micro-molar plasma concentration at which they have unacceptable cardiac and immunosuppressive side-effects, respectively (67, 68). The recently optimized P-gp inhibitors, tariquidar and zosuquidar, were designed for increased potency (10–100 nM) and enhanced specificity to P-gp (69–71). However, there seems to be some conflicting literature evidence on the role of tariquidar as an ATPase inhibitor or enhancer and if it is a substrate or inhibitor to P-gp (72, 73). Weidner et al., have recently reported that tariquidar is an inhibitor and nor substrate or both human and mouse P-gp (69).

**Table 1:** List of the known P-gp substrates and inhibitors.

**P-gp Substrates**	**P-gp Inhibitors**
Vincristine	Verapamil
Vinblastine	Cyclosporin A
Etoposide	Tamoxifen
Mitomycin C	Megestrol acetate
Paclitaxel	Quinine
Topotecan	Azodipin
Actinomycin D	Flupentixol
Doxorubicin	Valspodar
Daunorubicin	Dofequidar
Mitoxantrone	Tesmilifene
Epirubicin	Zosuquidar
	Tariquidar

Unlike enzyme-substrate interactions, such as lock-and-key model or induced-fit model, the tertiary structure of P-gp does not have a well-defined ligand-binding pocket making it innately perplexing to design highly specific competitive inhibitors for P-gp (74, 75). However, development of uncompetitive inhibitors for drug-binding pockets, and also, as ATP-binding NBD is well-characterized development of competitive inhibitors for NBD is feasible. Some bivalent inhibitors such as reversible dimer of quetiapine (74) and prodrug dimer of paliperidone (74), with ability to bind with multiple interaction sites of P-gp have been designed with limited success to enable better inhibition compared to monovalent binding (76). A drug design strategy to modify the already identified and natural compounds with known P-gp interaction to enhance specificity and potency will minimize the dose needed for clinical human application. Epothilones which have a chemical structure similar to taxanes, a microtubule mediated cell-division inhibitors, have been suggested to be substrates of P-gp with poor specificity (77). An analog of epothilone B, ixabepilone (azaepothilone B), in combination with capecitabine, was demonstrated success in treating anthracycline and taxane resistant metastatic breast cancer (78, 79). Further ixabepilone demonstrated a 6–10 fold higher cancer cell cytotoxicity compared to epothilone B, against a panel of over 20 tumor cell lines which included both drug-sensitive and resistant P-gp overexpressing cancer phenotypes (43, 80). Similarly, semisynthetic analogs of taxanes have been utilized in the development of several novel compounds with significantly higher efficiency against paclitaxel-resistant cancers. These include cabazitaxel (FDA approved) and ortataxel which have been shown to be efficient in hormone refractory metastatic prostrate cancer (81, 82). Further, these compounds have been shown to be less amenable to efflux by P-gp. Along with taxanes extensive studies have been performed on synthesizing analogs of vinca alkaloids. Vinflunine, a fluorinated semisynthetic analog of vinblastine, displayed 2–13 fold diminished susceptibility to efflux by P-gp-mediated compared to vincristine and vinblastine (83). Consequently, vinflunine received approval in Europe (2009) as second-line therapeutic agent against urothelial cancers. Similarly, an isoindoline urea derivative of vinblastine (at the same chemical position C20) was shown to possess 100 fold higher cytotoxic potential against vinblastine- resistant cancer cell lines (84, 85). Along the lines, an aryl amide derivatives of vinblastine has also been demonstrated to be less sensitive to P-gp mediated efflux in cancer cell lines (86). However, the therapeutic efficiency of these drugs is yet be prove in clinical settings.

As P-gp is considered to recognize hydrophobic compounds for efflux, adding a polar moiety to the drug by chemical modification of the drug or conjugating the drug with polar ligand could be considered some of the possible strategies toward reduced P-gp mediated efflux. Various nano-sized carriers and drug-conjugates have been studied to treat P-gp mediated MDR. Liposome-mediated doxorubicin delivery has received FDA approval in as early as 1995 (87). Other ionic and block copolymer-based drug modifications are still under study. An albumin-bound paclitaxel, abraxane, has already received FDA approval for treatment of metastatic breast cancers (88). Opaxio/Xyotax, a poly-L-glutamic acid- paclitaxel, is currently under Phase III clinical trials for the treatment of ovarian and esophageal cancers (89). Cell-penetrating macromolecules (CPMs) and antibody-drug conjugates (ADCs) have been extensively utilized in targeted drug delivery and reduce side-effects (90, 91). Octaarginine-conjugated taxol has been extensively studied in resistant cancers. These conjugation techniques were primarily intended to enhance drug internalization (92). However, cytoplasmic drug concentration and eventual impact on lowering P-gp mediated efflux by these conjugation techniques is not convincing. Gemtuzumab-ozogamicin was approved by FDA for a brief time-period, but later the approval was withdrawn due to lack of improved overall survival profile (93). Similarly, CD33-conjugated maytansine is efficient in pre-clinical studies, however, clinical benefit is yet to be proven (94). Brentuximab-vedotin with a potency to evade P-gp-efflux seems to be one of the very few ADCs which received FDA approval for treatment of refractory hodgkin's lymphoma and systemic anaplastic large cell lymphoma (95). As the understanding of conjugation techniques improve more efficient compounds could designed.

## P-gp Function in Tumor Immunity

The expression of P-gp on immune cells is shown to be correlated with immune cell activation, phenotype switch, and cytokine release. While expression of P-gp in peripheral circulating monocytes is extremely limited, however, its expression in tumor infiltrating anti-inflammatory MΦ2 tissue macrophages is extremely high (96). In dendritic cells, P-gp expression is correlated with their maturation and activation with enhanced professional antigen presenting functionality (97, 98). Blockade of P-gp with valspodar impaired DC maturation as shown by decreased expression of activation markers, CD80 and CD40 (98). Among all the innate immune cells, natural killer (NK) cells have been shown to have highest surface expression of P-gp, which is shown to correlate with the downstream cytotoxic functionality of these cells with enhanced Fas-mediated (Fas/FasL) surface binding of P-gp+NK cells to the target cells leading to release of inflammatory cytotoxic secretory granules leading to apoptotic death of target cell (99, 100).

The role of P-gp expression in adaptive immune cells varies with individual cell type. In B-cells, P-gp expression is correlated with cell migration and transitional phenotype in lymph nodes (101, 102). In CD4+T cells, P-gp is associated with inflammatory Th1/Th17 effector phenotype, while its expression is extremely limited in anti-inflammatory Treg phenotype (13, 103, 104). In CD8+T cells, the expression of P-gp is associated with memory (IL18Rα^+^CD161^+^CD62L^lo^) phenotype (105–107). These P-gp expressing CD8^+^ memory T cells in mucosal associated T-cells (such as in gastrointestinal tract) is associated with a bidirectional responses, with initial protective role to evade xenobiotic toxins, but later when normal microbiome is disrupted, could cause enhanced effector responses leading to autoimmune diseases such as crohn's disease and ulcerative colitis (107).

The immune cells in the patients with hematological malignancies, such as acute myeloid leukemia (AML), diffuse large B Cell lymphoma, multiple myeloma, and follicular lymphoma, demonstrate enhanced expression of P-gp, thus making these cancers resistant to chemotherapy (108, 109). Enhanced expression of P-gp in myeloid and lymphoid lineage cells of AML and B-cell lymphomas, respectively, is associated with upregulation of MAPkinase/ERK signaling (110). It is of interest to note that P-gp mediated chemoresistance could be overcome with monoclonal antibodies (mAb)-based anti-CD20 and anti-CD19 therapy, possibly because mAb could not be effluxed by P-gp (111).

To date there is very limited data from solid organ tumors showing P-gp expression in the infiltrating immune cells. Studies on human colorectal cancer demonstrated that there is enhanced frequency of P-gp expressing mucosal derived CD8+T cells in the tumor tissue specimens (112). However, the exact role of these CD8+T cells remains elusive. Further, the infiltration of the immune cells into the tumor could be skewed by the chemo-resistance of the cancer cells in the tumor microenvironment. In AML patients on long term chemotherapy there was enhanced CD4^+^CD161^+^P-gp^+^ T cells phenotype (113). Further this subset of CD4+helper-T-cells demonstrated diminished expression of T-cell exhaustion markers PD-1 and CTLA-4. The subsets of CD4+T-helper cells, Th17 and Th1 are known to induce anti-tumor effect through secretion of inflammatory cytotoxic cytokines such as IL-17, IFNγ, TNFα, and granzyme (114, 115). Interestingly, the tumor infiltrating P-gp-expressing CD4+T-cells (CD4+CD73+T cells) in breast and ovarian carcinomas were shown to exert enhanced secretion of these anti-cancer cytokines (116, 117). Importantly, chemical inhibition of P-gp inhibited vesicular secretion of these cytotoxic cytokines by these T-lymphocytes (14). Therefore, it will not be favorable to use P-gp-inhibitors in this scenario, as this might reduce the cytotoxic potential of these tumor infiltrating anti-cancer Th1 and Th17 CD4+T cell phenotypes (118). Further, as mentioned above P-gp expression is shown in pro-tumor MΦ2-macrophage phenotype and, anti-tumor NK-cell and Th17/CD4+T cell subsets, thus suggesting an apparently conflicting role of P-gp in tumor immunology. Therefore, the role of P-gp expression in tumor infiltrating immune cells should be more carefully studied in future to determine the potential application of combinatorial strategy of P-gp inhibitors with immune-checkpoint therapy (anti-CTLA4/anti-PD1) (119, 120).

## Conclusion

Despite of implementing multi-drug regimens, cancer therapy is still a challenge as tumor cells quickly develop resistance. The role of P-gp in chemo-resistance is well-appreciated for over past three decades. However, development of P-gp-specific inhibitors requires a better understanding of the tissue distribution, cell type specificity, body distribution/toxicity, immune side-effects, and cell-specific cytotoxicity. Synthetic modification of current chemotherapeutic drugs to evade P-gp-mediated efflux seems to be a very difficult drug-discovery task. Advances in the understanding of the 3D-crystal structure of P-gp protein offered novel insights into the drug-design strategies. To make the matters more complicated, adopting a combinatorial therapeutic regimen with P-gp inhibitors could enhance tumor cell drug-sensitivity, but impair efficient infiltration of tumors with anti-tumor immune cells. These changes in tumor microenvironment require further in-depth research for efficient futuristic usage of P-gp inhibitors.

## Author Contributions

KR and VT participated in manuscript drafting and revision of this review article.

### Conflict of Interest

The authors declare that the research was conducted in the absence of any commercial or financial relationships that could be construed as a potential conflict of interest.
